# Meaning of death among care workers of geriatric institutions in a death-avoidant culture: Qualitative descriptive analyses of in-depth interviews by Buddhist priests

**DOI:** 10.1371/journal.pone.0276275

**Published:** 2022-10-18

**Authors:** Yukan Ogawa, Akinori Takase, Masaya Shimmei, Shiho Toishiba, Chiaki Ura, Mari Yamashita, Tsuyoshi Okamura

**Affiliations:** 1 Institute of Regional Development, Taisho University, Tokyo, Japan; 2 Department of Psychology and Welfare, Den-en Chofu University, Tokyo, Japan; 3 Japan Society for the Promotion of Science, Tokyo, Japan; 4 Tokyo Metropolitan Institute of Gerontology, Tokyo, Japan; Jonkoping University, SWEDEN

## Abstract

**Objective:**

Care workers’ views of clients’ death have not been explored in Japan because of a cultural tendency to avoid talking openly about death. However, given the arising problems in end-of-life care settings, such as abuse and burnout, understanding care workers’ views regarding death is essential for designing effective interventions. We had two main research questions: Do care workers in Japan have their own ideas about death after working in the landscape of dying and death? Do these ideas influence care workers’ professional lives?

**Methods:**

We recruited interviewees based on a quantitative survey of care workers at 10 geriatric institutions in Tokyo. Among the 323 respondents, 23 survey respondents were willing to participate in an interview. After the scheduling process, nine individuals were able to participate in an in-depth interview.

To overwhelm cultural avoidance regarding death that prevents care workers from talking openly about death, Buddhist priests conducted interviews in the current study. Physicians and researchers assisted the interviews.

Because this was exploratory studies in which little is known about the topic in question, we adapted a qualitative descriptive approach.

**Findings:**

Thematic analysis revealed that: 1) care workers had clear views about conditions of clients’ good death after working in the field of dying and death; 2) care workers were motivated by past experiences of being close to dying themselves; and 3) care workers regarded their care for the dying as an experience that enriched their lives. In addition, the results revealed that the concept of spiritual care in Japan is still its infancy among care workers because of its vague definition.

**Conclusions:**

Care workers were willing to work for dying people with their view of death, and regarded their jobs as important opportunities for personal growth through caring for the dying.

## 1. Introduction

The low birthrate and aging population in Japan mean that society has become an enveloped one where death is omnipresent. The concept of a “death-ridden society”, or one where the death rate substantially exceeds the birth rate, is now a major concern in Japan [1,2]. It has traditionally been most common for people in Japan to die in hospital. From a longitudinal perspective, the percentage of people dying in hospitals in Japan increased from 10% in 1951 to 80% in 2000 [3]. However, because of the number of older people requiring long-term care, geriatric institutions are becoming an increasingly common place of death. The Japanese government has established special payments for end-of-life care in nursing homes, and the number of deaths in nursing homes has slowly increased [4]. This has resulted in task-shifting of end-of-life care from hospitals to geriatric institutions.

However, a number of problems have been identified in geriatric institutions in Japan. First, the number of reported cases of elderly abuse in Japanese geriatric institutions increased almost fivefold within 10 years, from 451 in 2008 to 2,187 in 2018 [5]. Second, according to one survey of care workers in geriatric institutions [6], emotional exhaustion, depersonalization, and low personal accomplishment were reported by 51.6%, 31.4%, and 83.8% of respondents, respectively. According to a survey of all geriatric institutions in one prefecture, no institutions had implemented an end-of-life care manual, and 64.7% of surveyed care workers had no education regarding end-of-life care [7]. We suggest that one of the reason of abuse and burnout is that staff is not adjusted to the task-shifting which is loading heavy burden for the staff. For designing effective interventions, understanding care workers’ views regarding death is essential.

There is a cultural barrier in investigating view of death in Japan. From an anthropological perspective, death is traditionally not spoken about in Japanese society, functioning as a cultural barrier called kegare [8,9] This tradition has roots in ancient Japan and a background in Shinto-Buddhist relations with a complex history of coexistence [10,11]. Our clinical experiences suggest that this avoidance also acts as a driving force in geriatric institutions to keep death out of sight. For example, until recently, when nursing home residents were known to be dying, it was a relatively common practice to transport them to hospital to die there. According to one report in 2014 [12], of the 147 cases of cardiopulmonary arrest that occurred in geriatric institutions in one city, emergency services were called in 80 cases and 51 cases were eventually taken to hospital (where they officially died). This finding suggests that even in cases of apparent death, it is considered preferable for incidents to be passed on to the medical system rather than being recorded at geriatric institutions.

In Japan, view of death have been studied mostly in the context of palliative care. For example, the Good Death Inventory (GDI) [13] was developed for evaluating good death from the bereaved family members’ perspective in the palliative care units of cancer centers. This scale is used by palliative care medical staff to assess patients’ death retrospectively. However, although Tsuji et al [14] assessed views of death among care workers in the geriatric institutions using the Frommelt Attitude Toward Care Of Dying Scale, it have not been explored using in-depth interviews in Japan.

In view of the difficulties mentioned above, we decided to use interviewers who are best placed to talk about death. We had two main research questions: Do care workers in Japan have their own ideas about death after working in the landscape of dying and death? Do these ideas influence care workers’ professional lives?

## 2. Materials and methods

As mentioned above, cultural avoidance regarding death in Japan prevents care workers from talking openly about death. Buddhist priests conducted interviews in the current study, for two main reasons. First, Buddhist priests were able to encourage interviewees to talk openly about life and death because they are typically regarded as being accustomed to death. Second, having Buddhist priests as interviewers encouraged interviewees to talk about distress in their jobs because Buddhist priests are widely regarded as good listeners in a confessional context.

We adapted a qualitative descriptive approach in this study [15]. Qualitative description is useful in exploratory studies in which little is known about the topic in question. The aim of this approach in the current study was not theoretical development. Rather, because the interviewees had unique perspectives regarding their own experience, the researchers’ role was to analyze the interview data and identify themes in those data.

### 2.1. Sample

**Fig 1:**
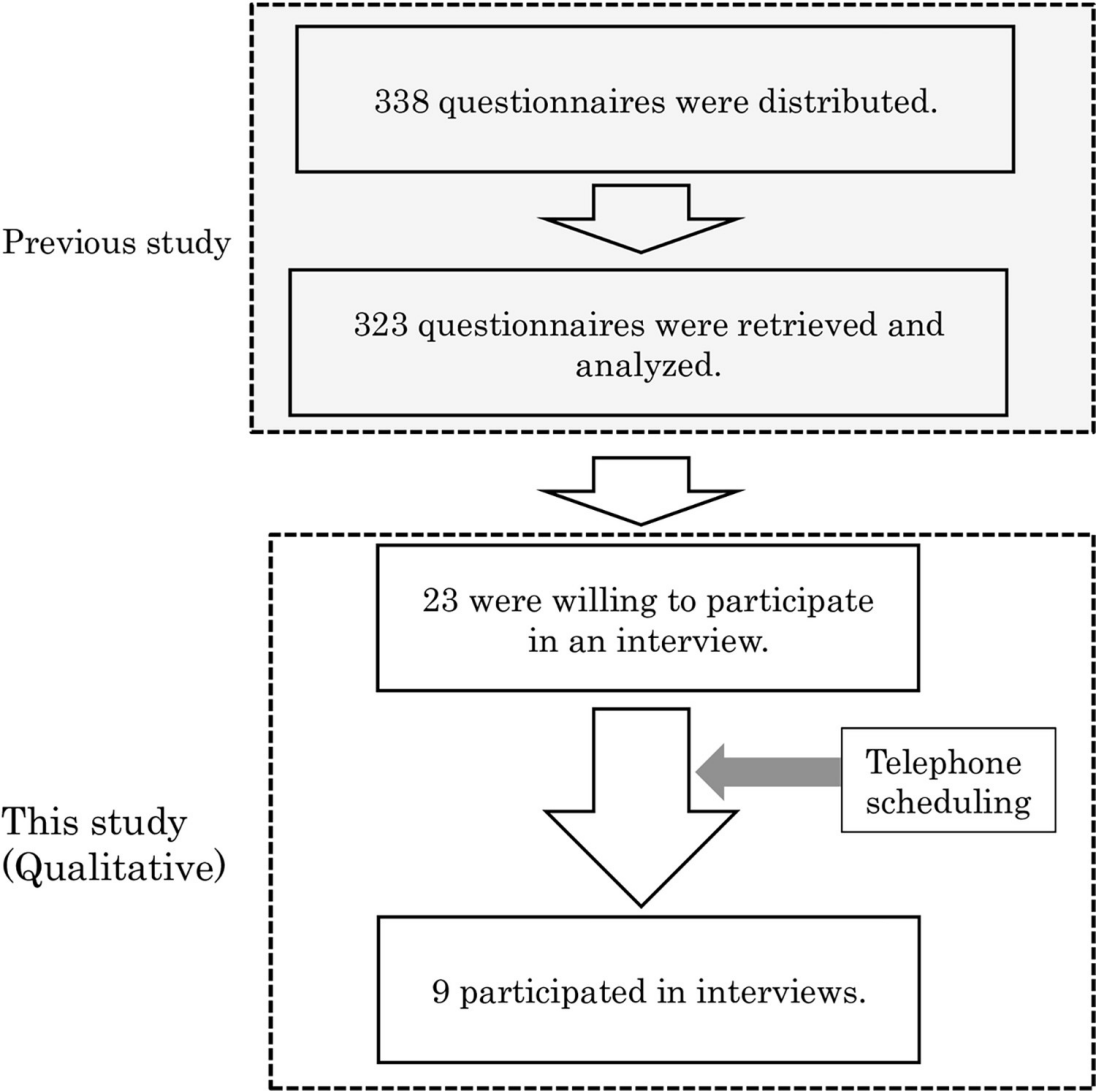
Flow of the study.

**Fig 2:**
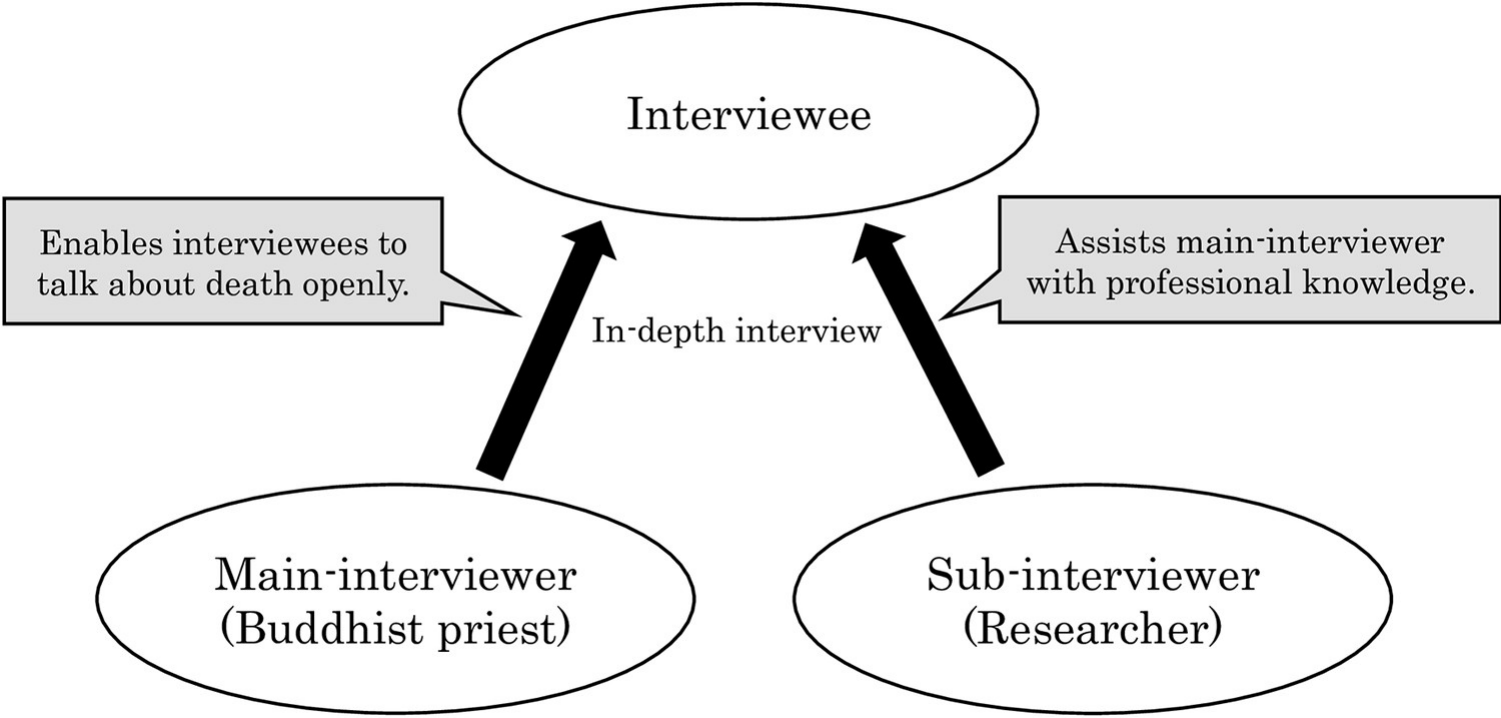
Interview method.

We recruited interviewees based on a questionnaire survey of care workers at 10 geriatric institutions within Metropolitan Tokyo carried out in 2017 [16]. Two of the institutions were hospitals with long-stay facilities until death for people with dementia, covered by public health insurance. Eight of the institutions were nursing homes for older people who require long-term care until the end-of-life stage. To recruit interviewees for the study, we invited all survey participants to also participate in in-depth interviews. Among the 323 (out of 338) respondents, 23 survey respondents were willing to participate in an interview. The interviews were scheduled to be held in February 2018 at Taisho University, located at Metropolitan Tokyo. After the scheduling process, nine individuals were able to participate in an in-depth interview. The flow of the study and interview methods are shown in Figs 1 and 2.

### 2.2. Procedures

We prepared a short interview guide comprising the items to be mentioned (Table 1). We attempted to create a laid-back atmosphere to reduce interviewees’ nervousness in front of the university teacher and Buddhist priest. During the interviews, the priests did not wear religious vestment. Interviewers listened attentively and did not respond with value judgments. The interviewers encouraged the interviewees to talk about their thoughts without any hesitation, using phrases like “Can you tell me more about it?” and “Thank you for sharing your interesting story with us.” The interviewers and interviewee sat in an L-shape without facing each other. We avoided a one-question and one-answer format for the interview, and instead adopted a relatively flexible interview format. For example, item 3 “their everyday work” usually started with the question “Please tell us about your work,” and the interviewers thoroughly listened to the interviewee’s narrative without any judgment. All of the participants spontaneously talked about life and death in this setting. The interview plan with this guide was tested with two care workers who were our study collaborators, and we received comments that no changes were necessary.

**Table 1:** Interview guide.

1. Basic information such as age, profession, length of time in the profession, and length of time working at their current institution 2. Brief personal history *Could you give me your brief biography*? *Where were you born*? *Please tell me about your hometown*. *Please tell me about your school life*. *Why did you choose this job*? 3. Details of their everyday work. *Tell us about your work*. *What happens during a usual work day*? 4. Positive and negative things about taking care of the elderly. *Please tell us what makes you feel happy when you are taking care of the elderly*. *Please tell us about what you find difficult about taking care of the elderly*. 5. Mindset regarding older adult care and death. *Why are you involved in care work*? *How do you feel about life and death as you do this work*? If the interviewee starts to talk freely, let them go with the flow.Listen without making value judgments, interviewers should speak as little as possible, and be careful not to make religious interpretations.

Interviews were conducted by three researchers: the main interviewer, a sub interviewer (non-priest researchers), and a note taker. The main interviewer was always one of the two Buddhist priests in our team (Y.O. and A.T.). All interviewers and interviewees were Japanese. Non-priest researchers had Catholic or Buddhist backgrounds. After introducing the research team and interview guidelines, interviewees were encouraged to talk about themselves. All interviewees appeared to be interested in the interviews with the priests, and spoke freely. The interviewees were allowed to request that audio recording was stopped temporarily if they felt that a topic was too personal to be analyzed by the researchers.

### 2.3. Data analysis

All interviews were conducted in Japanese, and were audio recorded and transcribed verbatim. Field notes were also made during the interviews. The transcripts were sent to participants and verified by them. After verification, the transcripts were read several times to allow us to become familiar with the data.

As mentioned in the Procedure section, we used a fairly flexible interview protocol, even though we had an interview guide which contained the items to be mentioned at least once. Because of the Buddhist priests’ listening ability, participants were able to talk a lot. However, the interview was not a question-and-answer session, and the topics went off on various tangents, with conversation flowing back and forth. Thus, in the analysis, diverse subjects were brought together.

Because the current study was an exploratory study of perspectives on death, as described by care workers at geriatric institutions, we sought to obtain an overall picture of care workers’ realities. Following the principles of qualitative description methodology, we conducted thematic analysis to identify themes in the data and establish meaningful categories concerning interviewees’ views regarding death and how their perspectives were influenced by their profession [17]. Text fragments were coded using MAXQDA 2018®. Constant comparisons were conducted. All of the authors had access to the whole data, and everything concerning the study was decided in the research team by unanimous consensus in meetings held twice a month.

All of the analyses were conducted in Japanese. After the manuscript was prepared in Japanese, it was translated into English by the authors and a native English-speaker. All authors agreed that the English version of the manuscript retained the meaning of the analyses.

### 2.4. Ethics approval and consent to participate

The study protocol was approved by the Ethics Committee of the Taisho University (17–001) on 25/07/2017. Written informed consent was obtained from all participants prior to the investigation. All participants signed a form declaring their informed consent for the results of the study to be published.

## 3. Results

All interviews were conducted without any issues and took approximately 90 minutes to complete. The characteristics of interviewees are shown in Table 2. In addition, the aspects of religiosity of the interviewees that were willingly shared during the interview are shown in Table 3, indicating that although some interviewees mentioned religion-based behavior, none of the interviewees declared their religious beliefs during the interview.

**Table 2:** Characteristics of interviewees.

Subject	Gender	Age	Profession	Length of time in the profession (years)	Type of institution	Other career in the area of caring
1	F	40s	Nurse	23	Nursing home	NICU
2	M	50s	Nurse	20	Geriatric hospital	General hospital (diabetic unit, stroke unit)
3	M	40s	Care manager	18	Nursing home	None
4	M	40s	Care manager	10	Nursing home	None
5	F	50s	Psychiatric social worker	10	Geriatric hospital	Welfare consultant in several geriatric institutions
6	M	Not recorded	Qualified care worker	8	Geriatric hospital	None
7	F	Not recorded	Qualified care worker	Not recorded	Geriatric hospital	Clerk of the group home
8	F	Not recorded	Care manager	10	Geriatric hospital	Geriatric institution
9	F	30s	Psychologist	12	Geriatric hospital	None

**Table 3:** Summary of participants’ religiosity disclosed in the interview.

1	Her grandmother was on a Buddhist pilgrimage and she once followed her grandmother’s journey. According to the interviewee, she wasn’t looking for anything and didn’t find anything.
2	He doesn’t belong to a specific Buddhist denomination. However, he is interested in history and religion. At the end of the interview, he happily talked about the origin of several denominations from a historical viewpoint and exhibited knowledge of Buddhist art.
3	He didn’t talk about his religiosity.
4	After 40, he started to read books on religion and ethics.
5	Her family’s grave is in their temple but she only goes there once a year. She says she has no religious beliefs herself.
6	He said only that he was a Buddhist.
7	She used to put her hands together to pray to the Buddha and the deceased when she was a child, but she stopped doing so some time ago. She said that this was because she began to think that the living were more important.
8	She says she is an atheist but respects other people’s beliefs
9	She goes to the temple to visit her family grave, but says that she doesn’t actually believe in anything.

*Note*. Although the interviewer did not mention religiosity, most participants willingly talked about it. Because we did not examine religiosity in depth, we do not have comprehensive information.

### 3.1 Distance from death is different between hospitals and nursing homes

Interviewees reported that, in hospitals, when a patient died and other patients asked what had happened, the real reason was often not disclosed because of doctor-patient confidentiality. Conversely, in cases in which a resident had died in nursing homes and other residents asked what had happened, the real reason was disclosed. In addition, interviewees felt that death is never considered a good thing in hospitals, or is perceived as a defeat.

[In the nursing home] We clearly say that the person has died. We say that they went to a place that you and I will go some day. We never hide it. (No. 1)

The aim of medicine is to get people well and out of hospital, so it’s not surprising that dying is not considered a good thing in hospitals. (No. 2)

In the completely artificial world of the hospital, death is considered to be a defeat. (No. 3)

[In the hospital] We cannot talk about what happened to them because of the duty of confidentiality. (No. 9)

### 3.2. The instability of the concept of spiritual care in Japan

Although the interviewers did not mention “spiritual care” or “spirituality” because of the vague definition of these terms, some interviewees mentioned these words, as shown below.

…I’m afraid I don’t know what to do, even though I know the word “spirituality.” In practice, I wonder what spirituality means to the patient. (No. 2)

…My boss talks about spiritual care in relation to physical pain and whole-person pain, but I don’t know what spiritual care is. I think this word does not fit with the Japanese language. (No. 5)

…I’m not familiar with the words God or Buddha, or spiritual or mystic… (No. 3)

### 3.3. Condition of good death expressed by care workers

Care workers talked about conditions that enable a good death for older people who are dying.

*Death after an appropriate length of time*

[When an 82-year-old woman died] they said that it was an appropriate length of life…I was sad at her last moment, but [just after her death] we were all able to talk about the deceased and smile together, which was a good thing. (No. 5)

*Death with acceptance and gratitude*

The period of end-of-life care is also a time for the family to prepare for the death of their loved one. This preparation time allows the family and the staff to accept death and the reality that the patient is leaving. (No. 6)

Many people express gratitude at the last moment. When you know that there is no time left and you’re looking back, all you can express is gratitude. (No. 1)

*Death as their own*

A man has the power to be born, so I think a man has the power to die in their last moment. (No. 1)

[They are] Becoming frail as the candle going out. It is quite natural to die quickly. (No. 5)

The last moment was natural and clean, and they were not suffering from the pain. (No. 3)

*Death with family around*

We are not the people that the dying person wants to stay with them in their final moments. Even though they might not have a good relationship with their family or may not have been in touch for a long time, they always want their family to be there. (No. 3)

[It is preferable that] families come and stay and feed the dying person. They bring old photos and the room is full of loved ones. (No. 4)

### 3.4. Influence of death on the reason for care workers’ choice of work

According to some interviewees, there was a strong influence of death over their own lives. The interviewees talked about past experiences of being close to dying themselves, which led them to their work. Table 4 indicates themes revealed in our interviews concerning care workers’ view of death. The themes indicated in Table 4 have also been combined with in-text quotations to form a supplementary table (S1 Table), which has been restated.

**Table 4:** Themes revealed in our interviews concerning care workers’ view of death.

Conditions of a good death expressed by care workers	Death after an appropriate length of time
	Death with acceptance and gratitude
	Death as their own
	Death with family around
Influence of death on the care workers’ choice of work	Experience of being taken care of near death
	Experience of having cared for a dying loved one
Influence of death on care workers’ present lives	Opportunity for personal growth through deep communication
	Opportunity to rediscover of the holiness of life

*Experience of being taken care of after a near death experience*

[When I was a college student] I fell onto the railway tracks at the train station and I broke three ribs…after discharge, I had to ride my motorcycle because I had a part-time job, and I was hit by a car…I came near to death twice in one month, and I had to learn to live using a wheelchair with help from care workers. (No. 2)

*Experience of having cared for a dying loved one*

I think my experience of end-of-life-care of my grandmother at my home had a strong impact on me. (No. 5)

### 3.5. Influence of death on care workers’ present lives

The interviewees also talked about positive aspects of being involved in care recipients’ death.

*Opportunity for personal growth through deep communication*

I have seen a number of people die, and I feel I have changed as a person. I feel that this has been a meaningful, important process for my growth. (No. 5)

They [the dying older residents] are often strangers. If it is a short case, they may have moved to the institution just 2 or 3 weeks ago. But I never feel like that. I always feel like I have known them for a long time; 2 or 3 weeks can feel as if it was 2 or 3 years. In the very last few hours or few days, the period we were together suddenly feels like a long time. (No. 3)

In the institution, I had enough time to deeply think about what it means to die in the way that a person should. (No. 1)

*Opportunity to rediscover of the holiness of life*

I think that it is sacred to see a person who is leaving this life, who is born with love and gives back love to the people around them, and who is being loved at the last moment. (No. 1)

Because many clients die after living a long life, [I think that generally] death is not horrible or painful. [The reason is that] It is the result of having lived a hard life. (No. 9)

The day before the death, some people talk with the dead, such as his or her own grandpa or grandma…There is a biological line between the living and the dead, but there is also an invisible linkage. (No. 5)

## 4. Discussion

First, according to care workers, death was not talked about openly in hospitals because of explicit restrictions related to doctor-patient confidentiality, and possibly because death is regarded as a bad thing or a defeat in the hospital setting. Conversely, death was openly talked about in nursing homes. Although the current results cannot confirm that avoidance of death still exists in hospitals, our findings indicate that the distance from death differs between hospitals and nursing homes. This is also suggested by the difficulty of implementing dignity therapy in Japanese cancer centers. Akechi et al. [18] reported a high refusal rate in a feasibility study of dignity therapy for terminally ill cancer patients in 2012. After 19 patients (86%) refused to participate in the study among 22 eligible patients who had been admitted to two palliative care units, the research committee decided to stop consecutive sampling. The reasons for patients’ refusal included the following: “It just makes me think about death” and “Why would you recommend such a thing to me when I am dying?” These findings indicate that death is not easy to talk about in hospitals, even in recent years.

Second, concerning spiritual care, although we did not use this term in the interviews, some interviewees spontaneously used this language, but stated that they did not understand its meaning. A concept that did not originally exist in the Japanese language but was adopted from English is typically expressed as the phonogram “su-pi-ri-charu ke-a.” This word is beginning to be widely used as a phonogram in the field of care [19]. However, a lack of clarity regarding the concept of “spiritual care” in the Japanese context was also indicated in the current study. Interviewees appeared to be confused by this word, which is now starting to be used by management at care facilities, feeling that their perspectives were not appropriately represented by the idea of the term “spiritual care.” Further discussion among care workers and researchers, including religious researchers, gerontologists, end-of-life researchers, and psychiatrists, may be beneficial for clarifying the situation.

Third, interviewees regarded their jobs as important opportunities for personal growth. According to interviewees, caring for a dying person provides a chance to know a person deeply, which was reflected in the comment “2 or 3 weeks felt as if [it was] 2 or 3 years.” We speculate that this is because caring for a dying person is one the deepest form of communication between two mortal human beings. In end-of-life care, carers also reported seeing the holiness of life. Death was not necessarily a fearful prospect for the interviewees because they had learned from those for whom they cared that death is not just the end, but something more. This was referred to as a positive aspect of their job, which they felt was not often encountered in normal life.

Fourth, on the concept of a ‘good death’, we noted differences between our findings and GDI. This was developed from the bereaved family members’ perspective and is used by palliative care medical staff to make a retrospective assessment of patients’ deaths. GDI has 10 core domains, “Environmental comfort”, “Life completion”, “Dying in a favorite place”, “Maintaining hope and pleasure”, “Independence”, “Physical and psychological comfort”, “Good relationship with medical staff”, “Not being a burden to others”, “Good relationship with family”, and “Being respected as an individual”. Only the concept of “Death with family around” from our study is included in the GDI’s core domains. This significant difference might be because of differences between the perspectives of families of terminal cancer patients and care workers in geriatric institutions, or differences between the interviewers.

Finally, our literature search revealed that the views of care workers of the geriatric institutions have not been sufficiently explored to date although the view of death have been explored in the palliative care of the cancer patient. Hence, the mechanisms by which care workers understand the deaths of those they care for has been paid little societal attention. In the current study, the interviewees talked about why they volunteered to take part in this study. According to one interviewee, their workplace was a good place to ponder on a good life. Another interviewee reported that they often questioned their own inner world after experiencing a client’s death. The current results suggest that, when creating an intervention program for care workers, we should consider: 1) respecting the views of death among care workers as identified in this study; 2) linking work in geriatric institutions to staff members’ personal experiences of death; and 3) having a third party, such as a religious leader, act as a facilitator.

### Limitations

Our study involved several limitations that should be considered. First, because we focused on a largely unspoken issue that has not been adequately investigated to date, previous literature was not comprehensive. Second, because we did not conduct control interviews by non-Buddhist priests, the effect of the interviewer being a Buddhist priest is not clear. Third, only 23 (7%) of the 323 respondents who returned questionnaires were willing to be interviewed. There is therefore a risk that only the engaged caretakers volunteered. Fourth, this was an exploratory study and the number of people we could interview was pre-determined. We are not in a position to judge whether theoretical saturation was reached, and therefore used a qualitative descriptive approach. Finally, because of the nature of the thematic analysis, we were not able to investigate the mechanisms by which working in the landscape of dying and death cultivated clear views about death, which leads care workers to experience growth and develop a better understanding about who they are.

### Recommendations for further research

According to Walter’s reflections on death [20], understanding how to die well from slow degenerative diseases of old age is currently a major challenge. Further research at geriatric institutions is essential for moving toward societies in which a good death is universally delivered for frail older people.

Care workers were strongly influenced by their past experiences, which resulted in their decision to work in a landscape featuring death and dying. Interviewees were clearly willing to work for dying people, reporting that their choice of occupation was influenced by their experiences. However, previous studies suggest that they do not have opportunities to discuss whether their idea of a good death has been achieved. Some facilities hold a memorial service for deceased residents approximately once a year, but this is not publicized, and it is only possible to glimpse this in the facilities’ publications. Furthermore, the focus is on the bereaved families, not staff. The implementation of opportunities for staff to discuss ‘good deaths’ is a subject for future research.

Considering interviewees in this study talked openly in interviews conducted by a Buddhist priest, the current findings suggest that using Buddhist priests as mediators or facilitators to support people to talk openly about death may be a valuable method in medical or care settings. Japan has little history of chaplaincy, and Buddhist priests were traditionally not welcome in hospitals because they were linked with death and funerals by patients and families. Although this phenomenon is widespread, it has not been addressed in academic literature. In the newsletter of the Buddhism trans-denomination organization supported by 60 denominations that was founded in 1963 to serve children through Sunday schools [21], one priest reflected that he was initially rejected by patients, who used the words “I’m not dead yet!” and became depressed as a result. Although the issue of propagation might be a serious concern, more collaboration between care providers and religious workers should be explored to deliver a good death for clients.

Our results also suggest the instability of popular terms relating to spirituality in actual end-of-life care settings. To create a safe place for care workers to freely talk about death, introducing religious workers who are already integrated into society may be more practical than merely introducing new words regarding spiritual care.

## Supporting information

S1 Table Themes revealed in our interviews concerning care workers’ view of death and narratives of the care workers. (DOCX)
