# Supplementary material for: Meaning of death among care workers of geriatric institutions in a death-avoidant culture: Qualitative descriptive analyses of in-depth interviews by Buddhist priests
Source: PLoS One. 2022 Oct 18;17(10):e0276275. doi: 10.1371/journal.pone.0276275 (PMC9578581; doi:10.1371/journal.pone.0276275)
Supplement: S1 Table — (DOCX) [file pone.0276275.s001.docx]

S1 Table. Themes revealed in our interviews concerning care workers’ view of death and narratives of the care workers

| Condition of good death expressed by care workers | |
| --- | --- |
| Death after appropriate length of time | |
|  | [When an 82-year-old woman died] they said that it was an appropriate length of life…I was sad at her last moment, but [just after her death] we were all able to talk about the deceased and smile together, which was a good thing. (No. 5) |
| Death with acceptance and gratitude | |
|  | The period of end-of-life care is also a time for the family to prepare for the death of their loved one. This preparation time allows the family and the staff to accept death and the reality that the patient is leaving. (No. 6) Many people express gratitude at the last moment. When you know that there is no time left and you’re looking back, all you can express is gratitude. (No. 1) |
| Death as their own | |
|  | A man has the power to be born, so I think a man has the power to die in their last moment. (No. 1) [They are] Becoming frail as the candle going out. It is quite natural to die quickly. (No. 5) The last moment was natural and clean, and they were not suffering from the pain. (No. 3) |
| Death with family around | |
|  | We are not the people that the dying person wants to stay with them in their final moments. Even though they might not have a good relationship with their family or may not have been in touch for a long time, they always want their family to be there. (No. 3)  [It is preferable that] families come and stay and feed the dying person. They bring old photos and the room is full of loved ones. (No. 4) |
| Influence of death on the reason care workers choice of work | |
| Experience of being taken care of near death | |
|  | [When I was a college student] I fell onto the railway tracks at the train station and I broke three ribs…after discharge, I had to ride my motorcycle because I had a part-time job, and I was hit by a car…I came near to death twice in one month, and I had to learn to live using a wheelchair with help from care workers. (No. 2) |
| Experience of having cared for a dying loved one | |
|  | I think my experience of end-of-life-care of my grandmother at my home had a strong impact on me. (No. 5) |
| Influence of death on care workers’ present lives | |
| Opportunity for personal growth through deep communication | |
|  | I have seen a number of people die, and I feel I have changed as a person. I feel that this has been a meaningful, important process for my growth. (No. 5) They [the dying older residents] are often strangers. If it is a short case, they may have moved to the institution just 2 or 3 weeks ago. But I never feel like that. I always feel like I have known them for a long time; 2 or 3 weeks can feel as if it was 2 or 3 years. In the very last few hours or few days, the period we were together suddenly feels like a long time. (No. 3) In the institution, I had enough time to deeply think about what it means to die in the way that a person should. (No. 1) |
| Opportunity to rediscover of the holiness of life | |
|  | I think that it is sacred to see a person who is leaving this life, who is born with love and gives back love to the people around them, and who is being loved at the last moment. (No. 1) Because many clients die after living a long life, [I think that generally] death is not horrible or painful. [The reason is that] It is the result of having lived a hard life. (No. 9) The day before the death, some people talk with the dead, such as his or her own grandpa or grandma...There is a biological line between the living and the dead, but there is also an invisible linkage. (No. 5) |
